# Group B *Streptococcus* Sequence Type 283 Disease Linked to Consumption of Raw Fish, Singapore

**DOI:** 10.3201/eid2211.160252

**Published:** 2016-11

**Authors:** Priyanka Rajendram, Win Mar Kyaw, Yee Sin Leo, Hanley Ho, Wen Kai Chen, Raymond Lin, De Partha Pratim, Hishamuddin Badaruddin, Brenda Ang, Timothy Barkham, Angela Chow

**Affiliations:** Tan Tock Seng Hospital, Singapore (P. Rajendram, W.M. Kyaw, Y.S. Leo, H. Ho, W.K. Chen, D.P. Pratim, B. Ang, T. Barkham, A. Chow);; National Public Health Laboratory, Singapore (R. Lin); Ministry of Health, Singapore (H. Badaruddin)

**Keywords:** group B streptococcus, streptococci, Streptococcus agalactiae, sequence type 283, bacteria, outbreak, raw fish, raw foods, food-borne infections, food safety, Singapore

## Abstract

An outbreak of invasive group B *Streptococcus* (GBS) disease occurred in Singapore in mid-2015. We conducted a case–control study of 22 adults with invasive GBS infections during June 21–November 21, 2015. Consumption of raw fish was strongly associated with invasive sequence type 283 infections, but not with non–sequence type 283 infections.

Group B *Streptococcus* (GBS) disease is caused by *S. agalactiae*, a commensal bacterium that can be isolated from genitourinary and gastrointestinal tracts of up to 30% of healthy adults. GBS can cause skin and soft tissue infections, urinary tract infections, bacteremia, and meningitis in adults; pregnancy-associated infections can lead to invasive disease in newborns (*1*).

In mid-2015, an outbreak of invasive GBS disease was reported in acute-care hospitals in Singapore. An increase in GBS bacteremia rate was observed for all adult acute-care hospitals, including Tan Tock Seng Hospital (TTSH), a 1,600-bed adult tertiary-care hospital in central Singapore, which reported an increase in GBS bacteremia rate from 3.5 cases/month in January–December 2014 to 6.5 cases/month in January–June 2015. Some infected men and nonpregnant women reported consuming raw fish before infection.

On the basis of detection of GBS in fish samples during joint investigations by the National Environmental Agency, the Agri-Food and Veterinary Authority of Singapore, and the Ministry of Health, sales of raw fish dishes containing Asian bighead carp and snakehead fish were suspended on July 24, 2015 (during epidemiologic week 29) (*2*). Concurrently, serotyping of GBS isolates collected from hospitals showed an increase in serotype III, and multilocus sequence type 283 (ST283) was isolated from a patient with GBS meningitis (*1*). We conducted a study at TTSH to compare the epidemiology of invasive ST283 and non-ST283 infections and to assess factors associated with invasive ST283 infections.

## The Study

We conducted a prospective case–control study (case:control ratio 1:3.5) of patients admitted to TTSH during June 21–November 21, 2015 (epidemiologic weeks 25–46). Using a standardized, interviewer-administered questionnaire, we collected information on food exposure history, in particular consumption of raw or undercooked fish, beef, eggs, and vegetables, during the 2 weeks before admission. Information on demographics, clinical history, and laboratory results were obtained from medical records. All GBS isolates were sent to the National Public Health Laboratory and serotyped by PCR (*3*) and genotyped by multilocus sequence typing using a standard *S. agalactiae* strain (*4*). 

We defined case-patients as inpatients at TTSH during epidemiologic weeks 25–46 who had laboratory-confirmed GBS infections detected in samples from any sterile site (blood, synovial fluid, cerebrospinal fluid) (*5*) within 48 hours after admission (*6*). Controls were defined as inpatients at TTSH with negative culture results for any sterile site (i.e., without invasive disease) within 48 hours after admission. We compared characteristics, food and nonfood exposures, and clinical presentation of persons infected with ST283 with those infected with non-ST283 and controls (Table 1). We constructed a multivariable logistic regression model based on major variables of interest from univariable analysis to assess for independent factors associated with ST283 and non-ST283 infections (Table 2).

**Table 1:** Characteristics of patients with invasive group B *Streptococcus* ST283 or non-ST283 infections and controls, Singapore*

Characteristic	ST283, n = 9	Non-ST283, n = 13	Controls, n = 76	Comparison and p value		
				ST283 vs. non-ST283	ST283 vs. controls	Non-ST283 vs. controls
Demographic						
Sampled during epidemiologic wk 25–29	8 (88.9)	3 (23.1)	37 (48.7)	**<0.01**	**0.02**	0.09
Median age, y (IQR)	59.4 (25–70)	74 (46–88)	77.5 (42–99)	**0.03**	**0.002**	0.37
Age < 65 y†	7 (77.8)	3 (23.1)	18 (23.7)	**0.01**	**<0.001**	0.96
Female sex†	6 (66.7)	6 (46.2)	45 (59.2)	0.34	0.67	0.38
Chinese ethnicity†	9 (100.0)	11 (84.6)	58 (76.3)	0.22	0.10	0.51
Concurrent condition						
Diabetes mellitus†	1 (11.1)	5 (38.5)	33 (43.4)	0.16	0.06	0.74
Malignancy†	1 (11.1)	1 (7.7)	11 (14.5)	0.78	0.78	0.51
Cardiovascular disease†	1 (11.1)	4 (30.8)	29 (38.2)	0.28	**<0.01**	0.61
Renal disease†	1 (11.1)	2 (15.4)	25 (32.9)	0.77	0.18	0.20
Liver disease†	0	2 (15.4)	5 (6.6)	0.22	0.43	0.28
Gastrointestinal disease†	1 (11.1)	2 (15.4)	14 (18.4)	0.77	0.59	0.79
Respiratory disease†	0	2 (15.4)	16 (21.1)	0.22	0.13	0.64
Blood disorder†	0	1 (7.7)	28 (36.8)	0.39	**0.03**	**0.04**
Skin wound†	0	2 (15.4)	12 (15.8)	0.22	0.20	0.97
Charlson Comorbidity Score, median (range)	0 (0–9)	2 (0–7)	3.5 (0–12)	0.21	**0.03**	0.26
Any concurrent condition	3 (33.3)	11 (84.6)	69 (90.8)	**0.01**	**<0.001**	0.50
Hospitalized during 6 mo before admission†	2 (22.2)	6 (46.2)	31 (40.8)	0.25	0.28	0.72
Food history						
Ate raw or undercooked fish <2 wk before admission†	7 (77.8)	0	3 (4.0)	**<0.001**	**<0.001**	0.47
Ate raw or undercooked beef <2 wk before admission†	2 (22.2)	0	0	0.07	**<0.001**	NC
Ate raw vegetables†	1 (11.1)	0	4 (5.3)	0.22	0.48	0.40
Ate raw eggs†	0	0	13 (17.1)	NC	0.18	0.11
Other exposure history						
Fish-related activities (fishing, fish spa)†	0	0	0	NC	NC	NC
Clinical presentation						
Fever (temperature >38°C)	7 (77.8)	9 (69.2)	23 (30.3)	0.66	**<0.01**	**<0.01**
Musculoskeletal pain	7 (77.8)	4 (30.8)	20 (26.3)	**0.03**	**<0.01**	0.74
Fever and musculoskeletal pain	5 (55.6)	3 (23.1)	8 (10.5)	0.12	**<0.001**	0.20
<2 d between symptom onset and admission	4 (44.4)	11 (84.6)	39 (51.3)	0.05	0.70	**0.03**

*Values are no. (%) unless otherwise indicated. Bold indicates statistical significance. IQR, interquartile range; NC, not calculable. †Data collected by using questionnaire.

**Table 2:** Multivariable analysis of risk factors associated with invasive group B *Streptococcus* ST283 and non-ST283 infections, Singapore*

Factor	ST283			Non-ST283	
	aOR (95% CI)	p value		aOR (95% CI)	p value
Consumption of raw or undercooked fish <2 weeks before hospitalization	100.11 (6.21–1612.91)	**0.001**		NC	NC
Age <65 y	13.34 (1.14–156.56)	**0.04**		1.04 (0.25–4.27)	0.96
Charlson Comorbidity Score	1.02 (0.63–1.64)	0.95		0.85 (0.65–1.11)	0.23

*Bold indicates statistical significance. aOR, adjusted odd ratio; NC, not calculable; ST, sequence type.

A total of 22 case-patients (17 with bacteremia, 2 with septic arthritis, and 3 with bacteremia complicated by meningitis, epidural space abscess, and septic arthritis) and 76 controls (73 provided blood, 2 provided joint fluid, 1 provided both) were included in this study. Among 22 case-patients, 11 had serotype III GBS infections, of whom 9 had ST283 infections. None of the other serotype III strains, namely ST17 (*1*) and a new ST not previously reported (*1*), were single-locus variants of ST283. Other serotypes not typed were Ia (*3*), II (*5*), V (*2*), and VII (*1*).

Most (8/9) ST283 infections were identified during epidemiologic weeks 25–29, before suspension of sale of raw fish dishes. During epidemiologic weeks 30–46, only 1 ST283 case was identified, compared with 10 (77%) of 13 of non-ST283 cases. Persons infected with ST283 tended to be younger than persons infected with non-ST283 (median age 59.4 years vs. 74.0 years) (p = 0.033), and they were also less likely to have a preexisting medical condition (33.3% vs. 84.6%; p = 0.014). Seven (77.8%) of 9 ST283-infected patients had eaten raw or undercooked fish <2 weeks before admission, compared with 0 of non-ST283–infected patients (p<0.001) and 3 controls (p<0.001). Two of the ST283-infected patients who had eaten raw or undercooked fish had also eaten raw or undercooked beef. ST283-infected persons were more likely than controls to have high fever (temperature >38°C) and musculoskeletal pain (55.6% vs 10.5%; p<0.001); however, this comparison was not significant for persons with non-ST283–infection (23.1%; p = 0.12). Only 45 (60%) controls had a noninvasive infection.

Multivariate analysis showed that eating raw or undercooked fish during the 2 weeks before admission was independently associated with ST283 infection, but not with non-ST283 infection (Table 2), when compared with controls. None of the non-ST283–infected patients had eaten raw or undercooked fish during the timeline.

We also conducted a subanalysis that compared ST283-infected patients with onset up to epidemiologic week 29 with controls (who had similar infection onset and opportunity to purchase and eat raw fish). We observed a stronger association with eating raw fish among persons who could purchase raw fish dishes (adjusted odds ratio 12,423.62, 95% CI 1.51 to 1.02 × 10^8^; p = 0.04). However, 2 of the ST283-infected patients did not report eating raw fish, which suggests other means of acquiring ST283 infections.

## Conclusions

Several population-based studies have raised concerns about the increasing incidence of invasive GBS disease in men and nonpregnant women (*7*,*8*). We report an outbreak of invasive GBS serotype III ST283 infections in men and nonpregnant women. Molecular epidemiology studies have showed that ST283 strains from fish in Asia had the same virulence gene profile as human invasive isolates, which suggests potential exposure of humans and fish to common environmental sources of ST283 or transmission of the bacterium between different host species (*9*). Fish consumption was associated with an increased risk for acquisition of GBS in a prospective cohort study in the United States (*10*). However, data are limited for the likelihood and routes of interspecies transmission of this strain associated with fish and invasive disease in humans.

We found a strong association between consumption of raw or undercooked fish and invasive ST283 infections in men and nonpregnant women. Being older and having severe concurrent conditions were negatively associated with infection, contrary to findings in previous studies (*11*,*12*). Our findings suggest that non-ST283 strains caused symptomatic infection in more susceptible populations (those with more concurrent conditions), whereas ST283 might be more invasive and affected less susceptible persons.

An additional contributing factor could be differences in food consumption in local populations; elderly persons and those with severe concurrent conditions might avoid eating raw or undercooked foods (*13*). The finding that no non-ST283–infected patients had eaten raw or undercooked fish before admission strengthened our hypothesis of a link between the ST283 and consumption of raw or undercooked fish.

After isolation of ST283 from 4% of freshwater fish samples from restaurants, markets, stores, and fisheries, use of freshwater fish in all ready-to-eat raw fish dishes sold at retail food establishments was banned in Singapore on December 5, 2015 (*14*,*15*). The number of reported invasive GBS infections in Singapore has decreased, although sporadic invasive ST283 infections have been identified. TTSH has seen no new invasive ST283 infections identified since the study ended on November 21, 2015, despite continued active case-finding.

The relatively small number of ST283 and non-ST283 infections in this study is a limitation that precludes drawing definitive conclusions. However, isolation of ST283 from fish samples, the strong association of raw fish consumption with ST283 infection, and the sharp decrease of ST283 infections after the food ban strongly suggest a food-related outbreak. Although the outbreak of GBS disease in Singapore has been controlled, further studies on the virulence, transmissibility, and epidemiology of ST283 and risk factors are warranted to better manage future infections.
